# Discovery of *Sclerotinia sclerotiorum* Hypovirulence-Associated Virus-1 in Urban River Sediments of Heathcote and Styx Rivers in Christchurch City, New Zealand

**DOI:** 10.1128/genomeA.00559-13

**Published:** 2013-08-08

**Authors:** Simona Kraberger, Daisy Stainton, Anisha Dayaram, Peyman Zawar-Reza, Christopher Gomez, Jon S. Harding, Arvind Varsani

**Affiliations:** School of Biological Sciences, University of Canterbury, Christchurch, New Zealanda; Department of Geography, University of Canterbury, Christchurch, New Zealandb; the Waterways Centre for Freshwater Management, University of Canterbury, Christchurch, New Zealandc; Natural Hazards Research Centre, University of Canterbury, Christchurch, New Zealandd; Electron Microscope Unit, Division of Medical Biochemistry, Department of Clinical Laboratory Sciences, University of Cape Town, Observatory, South Africae; Biomolecular Interaction Centre, University of Canterbury, Christchurch, New Zealandf

## Abstract

In samples of benthic and bank river sediments of two urban rivers in Christchurch city (New Zealand), we identified and recovered isolates of *Sclerotinia sclerotiorum* hypovirulence-associated virus-1 (SsHADV-1), a fungus-infecting circular single-stranded DNA virus. This is the first report of SsHADV-1 outside of China and in environmental samples.

## GENOME ANNOUNCEMENT

Most fungus-infecting viruses have RNA genomes (either single or double stranded). However, *Sclerotinia sclerotiorum* hypovirulence-associated virus-1 (SsHADV-1), which infects the plant pathogenic fungus *Sclerotinia sclerotiorum*, has a 2,166-nucleotide (nt) circular single-stranded DNA genome (with two open reading frames that are bidirectionally transcribed). The replication-associated protein (Rep) of SsHADV-1 shares significant sequence similarity with integrons found in fungal genomes and geminivirus Reps. There have been no reports or identifications of SsHADV-1 except for the single report from China by Yu et al. (1) and the single SsHADV-1 sequence deposited in GenBank (accession no. GQ365709). Nonetheless, subsequent to the discovery of SsHADV-1, various related but diverse viruses have been discovered from cassava leaves (2), badger feces (3), mosquitoes (4), and dragonflies (5). Here, we report the recovery of four SsHADV-1 viral isolates from urban river sediments from the South Island, New Zealand.

Benthic and bank river sediment samples from the Heathcote River (6 sites) and Styx River (5 sites) in Christchurch city (New Zealand) were collected in 2012. Twenty milliliters of sediment slurry from each site was mixed with 30 ml of SM buffer (0.1 M NaCl, 50 mM Tris-HCl [pH 7.4]) and centrifuged at 10,000 × *g*, and the supernatant was filtered through 0.45-µm and 0.2-µm syringe filters. Total viral DNA was extracted from these filtrates using the High Pure viral nucleic acid kit (Roche Diagnostics) and enriched using rolling-circle amplification (TempliPhi, GE Healthcare). Ten microliters of enriched viral DNA from each sample was pooled (the two river samples were pooled separately) and sequenced at the Beijing Genomics Institute (Hong Kong) using the Illumina HiSeq 2000 platform. The resulting short paired-end reads were assembled using ABySS 1.3.4 (6). Through a BLAST (7) analysis of the assembled contigs (>1,000 nt), we identified a contig of 2,166 nt with 99% genome-wide pairwise identity toSsHADV-1 (GenBank accession no. GQ365709). Based on the sequence of the contig, we designed back-to-back primers (SR1-Scl-F, 5′-GATATTATACAAGGCGGTCAGGG-3′, and SR1-Scl-R, 5′-CATTACATAATAATCTCCCATACCTGCC-3′) to verify the genome and to screen and recover SsHADV-1 from all sediment samples. Using PCR amplification with HiFi HotStart DNA polymerase (KAPA Biosystems), four genomes (2,166 nt each) were recovered, one isolated from the Heathcote and three from the Styx River samples. These samples were then cloned and sequenced by primer walking.

The four genomes (GenBank accession no. KF268025 to KF268028) share >99.2% genome-wide pairwise identity and 98.1 to 98.7% pairwise identity with the SsHADV-1 isolate from China. The discovery of SsHADV-1 in river sediment samples indicates that these viruses are shed into waterways and reveals that SsHADV-1 has a larger global distribution. The possibility also exists that *S. sclerotiorum* found within the surrounding ecosystems of the Heathcote and Styx rivers in New Zealand is being mobilized by waterways, and with it, SsHADV-1. SsHADV-1 confers hypovirulence to *S. sclerotiorum* (1), and with recent studies by Yu et al. (8) showing the ease of extracellular transmission, the SsHADV-1 found in the two river systems in New Zealand may provide some biocontrol options for farmers in New Zealand to control the plant pathogenic fungus.

### Nucleotide sequence accession numbers.

The complete genomes of SsHADV-1 described in this report have been deposited at GenBank under accession no. KF268025 to KF268028.
